# A database of anti-coronavirus peptides

**DOI:** 10.1038/s41597-022-01394-3

**Published:** 2022-06-13

**Authors:** Qianyue Zhang, Xue Chen, Bowen Li, Chunying Lu, Shanshan Yang, Jinjin Long, Heng Chen, Jian Huang, Bifang He

**Affiliations:** 1 Medical College, Guizhou University, Guiyang, 550025 China; 2 School of Life Science and Technology, University of Electronic Science and Technology of China, Chengdu, 611731 China

**Keywords:** Databases, Bioinformatics

## Abstract

Since 2019, the novel coronavirus (SARS-COV-2) disease (COVID-19) has caused a worldwide epidemic. Anti-coronavirus peptides (ACovPs), a type of antimicrobial peptides (AMPs), have demonstrated excellent inhibitory effects on coronaviruses. However, state-of-the-art AMP databases contain only a small number of ACovPs. Additionally, the fields of these databases are not uniform, and the units or evaluation standards of the same field are inconsistent. Most of these databases have not included the target domains of ACovPs and description of *in vitro* and *in vivo* assays to measure the inhibitory effects of ACovPs. Here, we present a database focused on ACovPs (ACovPepDB), which contains comprehensive and precise ACovPs information of 518 entries with 214 unique ACovPs manually collected from public databases and published peer-reviewed articles. We believe that ACovPepDB is of great significance for facilitating the development of new peptides and improving treatment for coronavirus infection. The database will become a portal for ACovPs and guide and help researchers perform further studies. The ACovPepDB is available at http://i.uestc.edu.cn/ACovPepDB/.

## Abstract (summary)

**Table:** 

Measurement(s)	Inhibition effect of anti-coronavirus peptide
Technology Type(s)	Antiviral assay

## Background & Summary

The coronavirus disease 2019 (COVID-19) is triggered by a novel coronavirus called Severe acute respiratory syndrome coronavirus 2 (2019-nCoV) (SARS-CoV-2). COVID-19 has become a global public health major event^1^, causing an indelible impact on the global economy and human lives and health. Several approaches for fighting against coronaviruses, such as potential anti-coronavirus infection drugs and vaccines, have been reported^2,3^. However, there are only a few specific therapeutic drugs available to against coronavirus: the oral drug “Molnupiravir^4^”, neutralizing antibody “Sotrovimab^5^” that was recently approved by the Food and Drug Administration (FDA), antibody cocktail Casirivimab/Imdevimab (Ronapreve^TM^; REGEN-COV^TM^)^6^ which has no inhibitory effect on the variant “Omicron”, and BRII-196/BRII-198 combination therapy which was urgently approved by the National Medical Products Administration of China (NMPA)^7^. All of these drugs are only approved for emergency use.

The structural proteins of coronavirus contain four genera: spike protein (S protein), membrane protein, envelope protein, and nucleocapsid protein^1^. Among them, S protein mediates viral invasion by interacting with human angiotensin-converting enzyme 2 (ACE2) and dipeptidyl peptidase 4 (DPP4)^8^, which are the key proteins that determine the invasion of the virus^1^. And many potential anti-coronavirus agents target three primary domains of S protein: heptad repeat 1 domain (HR1), heptad repeat 2 domain (HR2), and receptor-binding domain (RBD). However, due to the increasing viral resistance, several existing antiviral drugs and therapeutics have unsatisfactorily inhibitory effects on coronaviruses^9–19^. Therefore, new antiviral drugs or treatment solutions are urgently needed to replace or supplement the currently used drugs.

Antimicrobial peptides (AMPs) are a family of compounds that have inhibitory effects on various types of microbial pathogens^20^. Part of peptides in AMPs are able to inhibit coronaviruses. These peptides are called anti-coronavirus peptides (ACovPs)^18^. Massive studies proved that ACovPs have an excellent ability to inhibit coronaviruses^18^. For example, P6, P8, and P10 exhibited anti-severe acute respiratory syndrome coronavirus (SARS-CoV) activities^21^. Li *et al*. discovered that Mucroporin-M1 has the property of decreasing SARS-CoV infectivity^22^. Moreover, a few ACovPs have been tested *in vivo* and showed delightful results^23,24^. Among them, 229E-HR2P was reported to effectively prevent HCov-229E infection in the mouse respiratory tract^23^. EK1, targeting the HR1 domain of S protein, showed its protective effect in the HCoV-OC43 and Middle East respiratory syndrome coronavirus (MERS-CoV) infection mouse models^24^.

Actually, copious scientists have devoted themselves to the identification and design of AMPs. Simultaneously, several AMP databases have been built and widely used in relevant scientific research. For example, Wang *et al*. established the eminent AMP database APD3 in 2016^25^, like dbAMP^26^ and DRAMP^27^, which contains rich information of AMPs, including amino acid sequence and inhibitory activity. Based on the data provided by these databases, predictive tools have been developed to identify novel ACovPs^28,29^. However, all of these databases contain only a small number of ACovPs. For example, AVPDB^30^ houses vast antiviral peptides but only 98 ACovPs. Additionally, the fields of these databases are not uniform, and the units or evaluation standards of the same field are inconsistent. Most of these databases ignore the following important information, including the target domains of ACovPs and description of *in vitro* and *in vivo* assays to measure the inhibitory effect of ACovPs. These drawbacks make it difficult for relevant scientific researchers to find the key information of ACovPs from these databases. Therefore, the anti-coronavirus community urgently needs a complete and comprehensive database to store experimentally validated ACovPs.

In this study, we developed the first database tailored for ACovPs storage and management, called ACovPepDB, which stored 214 unique ACovPs with 518 entries from published peer-reviewed articles and public databases. ACovPepDB is freely accessed online at without registration. All of the ACovPs data could be downloaded. This database is considered valuable in consolidating understanding of ACovPs and serves as a comprehensive resource for coordinating efforts to improve ACovP related research and stimulate data mining in bioinformatics.

## Methods

### Data collection

The purpose of ACovPepDB is to provide a comprehensive database for ACovPs. We collected information of ACovPs from published peer-reviewed articles and public databases.

To this end, an accurate text mining query to extract data of the latest information on ACovPs: “((((coronavirus) AND (peptide OR peptides) AND (inhibit* OR block*))))” has been used to search against the PubMed database. This search provided 2,199 peer-reviewed articles from 1972 to 2021 July. Meanwhile, ACovPs from other public databases, such as AVPDB^30^ and DPL^31^, were also curated into ACovPepDB.

### Data extraction

Information related to ACovPs was then manually extracted from related articles and databases, and curated in AcovPepDB. The information of source articles containing ACovPs data can be found in Supplementary Source Article. To ensure the comprehensiveness of data, data were collected according to the following criteria:

(1) ACovPs that do not contain sequences are not included in the database in principle.

(2) The detailed information of antiviral experiments of each entry is from the original articles, rather than from review papers.

(3) ACovPs from public databases were collected from published articles, which were revisited to extract other related information.

Eventually, a total number of 518 entries with 214 unique ACovPs were extracted and integrated into ACovPepDB.

### Database table design

ACovPs have inhibitory effects on coronaviruses. The aim of ACovPepDB is to provide the most comprehensive antiviral activity and related information of ACovPs. In ACovPepDB, the information provided by each entry contains a plurality of dimensions, among which the target domain is of great significance to the research of ACovPs. To help users retrieve the database clearly, data in ACovPepDB were stored into three database tables: “ACovpeptide”, “Targetdomain” and “Modification”.

Table “ACovpeptide” offers all the data related to ACovPs. Each entry includes the following information: ACovPid, peptide name, amino acid sequence or primary structure, peptide source, against virus, modification, similar peptide, inhibitory effects, assay description, target region name, reference article and three-dimensional structure.

The inhibitory effect of the ACovPs on coronaviruses is an indispensable factor. We deposited the inhibitory effect information of each ACovP in the following three fields: “InhibitionValueType”, “InhibitoryEffect” and “InhibitoryUnit”. The “InhibitionValueType” field represents the evaluation value types which were set as IC50, IC90, EC50, and IC100. The “InhibitoryEffect” field represents the exact value of the inhibitory effect. The “InhibitoryUnit” means the inhibitory effect units which were unified into μM and μg/ml. For example, the IC50 of an ACovP was measured to be <20 µg/mL. The values in “InhibitionValueType”, “InhibitoryEffect” and “InhibitoryUnit” fields of this ACovP should be “IC50”, “<20”, “µg/mL”. To explore the ability of ACovPs to suppress coronaviruses as completely as possible, the inhibitory effects of a certain ACovP on different viruses were recorded in multiple entries. We also designed a field “assay description” to describe the inhibition experiment process of ACovPs in each entry. This field can help users to quickly obtain the details of antiviral experiments and reproduce the inhibition experiment.

Each entry in table “ACovpeptide” has a field called “Similar Peptides”. This field displays the ACovPid of ACovPs most similar to the currently accessed ACovP, which allows users to fast and conveniently observe the correlation between ACovPs in ACovPepDB. The implementation of this field is based on ACovPBLAST, which is powered by the BLASTP (version 2.2.31+)^32^. The five ACovPs with the highest identity to the current peptide were displayed in this field, and users can click the corresponding ACovPid to jump to the detail page of the peptide.

ACovPepDB also provides structures of the peptides. However, only nine experimentally-determined peptide 3D structures have been curated in ACovPepDB, which made us to use the PEP-FOLD3^33^ and Phyre2^34^ server to predict structures of the rest natural peptides. PEP-FOLD3 was used to predict the three-dimensional structures of ACovPs with less than 50 amino acids, while Phyre2 was utilized to predict those of ACovPs with more than 50 residues. Structures can be visualized by the JSmol applet. Additionally, we designed a field named “StructureExperimentallyVerified” in Table “ACovpeptide” to indicate whether the corresponding ACovP structure model is experimentally verified or not.

The target region information of each ACovP was stored in the “Targetdomain” table. Each entry in “Targetdomain” includes the following information: entry name in Uniprot, target type, taxonomic identifier in Uniprot, synonyms, the source of target region, and the three-dimensional structure of target region.

The “Modification” table was designed for housing chemical modification information for modified ACovPs. Each entry in this table includes the following fields: ModificationID, Modification Name, Modified Peptide.

### Database framework

**Fig. 1:**
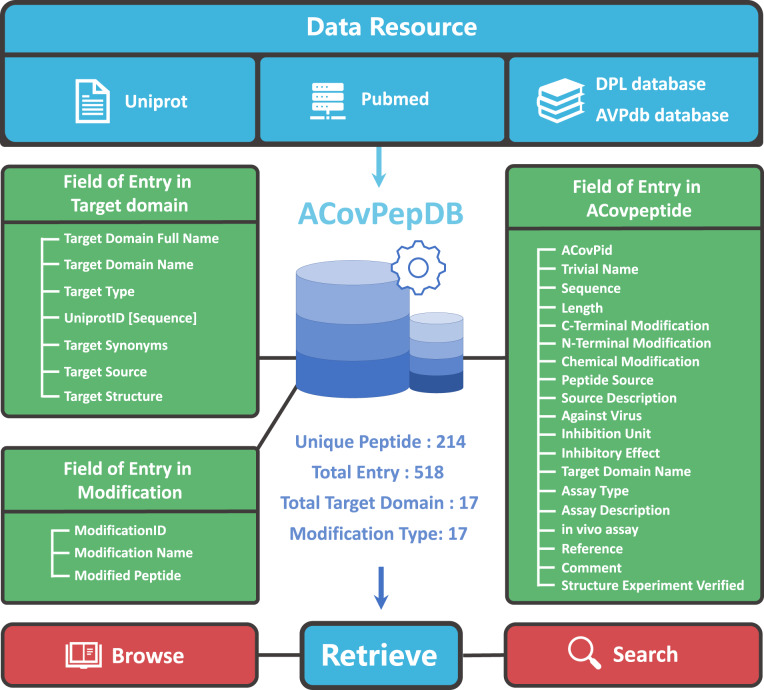
The overview of architecture of ACovPepDB.

The ACovPepDB database has been built based on a Linux-Apache-MySQL-PHP platform. All data were stored through the MySQL database as the back end, and the front end of the web interface was designed by using HTML, CSS, and JavaScript. Fig. 1 shows the architecture of ACovPepDB. ACovPepDB has three tables in the back end: ACovpeptide, Targetdomain and Modification. Table “ACovpeptide” provides all the information related to each ACovP. The data in the ACovpeptide table is organized in an assay-centered style. A peptide can be arranged into different entries if multiple antiviral assays were used to evaluate its inhibitory effect on various coronaviruses. Table “Targetdomain” offers the target region information of each ACovP, and table “Modification” holds the detailed information of each modification.

### Future plan

ACovPepDB will move with the times, not only continuing to update to deliver quality data, but also integrating tools to more easily interact with the data. The database is scheduled to be updated annually. We will keep curating data from the latest peer-reviewed published literature and related databases. Specifically, we are currently developing an artificial intelligence-based tool to predict and design novel ACovPs. The integration of such tools into ACovPepDB can accelerate the development of potential therapeutic candidates for treating coronavirus infection. Additionally, we will work with other public resources to make ACovPepDB more widely available. Sustained efforts will be made to improve the database, with the ultimate goal of facilitating anti-coronavirus research.

## Data Records

This database provided three datasets. The first dataset consisted of ACovP entries, which included ACovP name, amino acid sequence or primary structure, ACovP source, against virus of peptide, inhibitory effects, etc. The second dataset presented the entries related to target region, including the entry name in Uniprot, target type, taxonomic identifier in Uniprot, synonyms, etc. The last dataset was composed of the modification entries, including the ModificationID, Modification Name and Modified Peptide.

These datasets can be downloaded from the “download” web page of ACovPepDB^35^. The source data of ACovPepDB were also shared on Github and Gitee. And these datasets also can be downloaded from Figshare^36^.

In the current version of ACovPepDB, table “ACovpeptide” contains 214 unique ACovPs with 518 entries. Table “Targetdomain” includes 17 entries of target region. Table “Modification” possesses 17 types of modification.

## Technical Validation

**Fig. 2:**
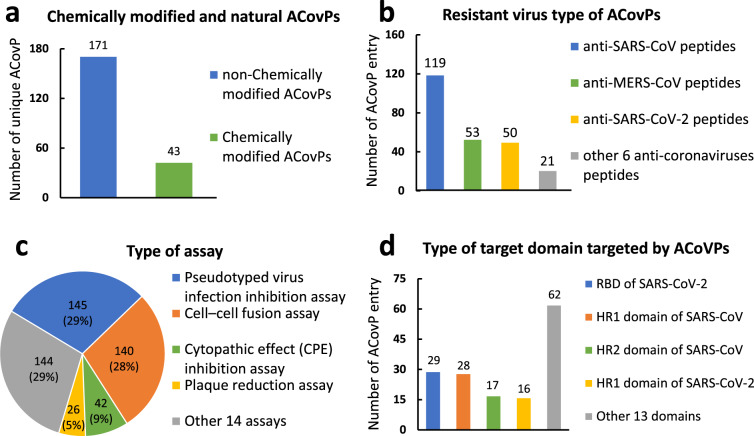
Distribution of (**a**) The chemically modified ratio of ACovPs, (**b**) The resistant virus type of ACovPs, (**c**) The type of assay, (**d**) The type of target domain. RBD (Receptor-binding domain), HR1 (Heptad repeat 1), HR2 (Heptad repeat 2).

After manually extracting, we integrated all ACoVPs into ACovPepDB. Summary of data entry can be found in Fig. 2.

### Table “ACovpeptide”

#### Chemically modified and natural ACovPs

As shown in Fig. 2a, these unique ACovPs can be divided into two categories: chemically modified or natural peptides, which contain 43 and 171 ACovPs, respectively.

#### Resistant virus type of ACovPs

The resistant coronaviruses of ACovPs can be classified into nine categories. As shown in Fig. 2b, 119 peptides can inhibit SARS-CoV, 53 peptides against MERS-CoV, 50 peptides restrain SARS-CoV-2, and 21 peptides against six remaining coronaviruses. Furthermore, researchers have explored the inhibitory effects of partial ACovPs on omnifarious coronaviruses. Through the statistics of ACovPepDB, 17 ACovPs are capable of inhibiting multiple coronaviruses.

#### Type of assay

In ACovPepDB, a total of 18 types of antiviral experiments were recorded. In order to verify the efficacy of peptides in multifarious assays, researchers conducted various antiviral experiments for a certain ACovP. Therefore, in the case of only 214 individual ACovPs in ACovPepDB, 497 entries have detailed information of anti-virus assay. As shown in Fig. 2c, the pseudotyped virus infection inhibition assay is mainly used which appeared in 29% of all entries. Next is the cell-cell fusion assay, which accounts for 28% of the total. These two assays are the most used, and the proportion of each other assay is less than 10%.

### Table “Target domain”

#### Type of target domain

S protein plays a role in the entry, binding, and fusion of coronavirus, which is a type I transmembrane glycoprotein of coronavirus^17^. ACovPepDB contains nine categories of resistant coronaviruses and 17 distinctive target domains. In this database, 152 entries with 104 unique ACovPs target to diverse domains of coronaviruses.

As shown in Fig. 2d, the target domain that is most targeted by ACovPs is the RBD of SARS-CoV-2, and 29 unique ACovPs are pointing to this domain. The second hottest target domain is the HR1 domain of SARS-CoV which is targeted by 28 individual ACovPs. And there are 17 unique ACovPs that target to the HR2 domain of SARS-CoV, 16 unique ACovPs target to the HR1 domain of SARS-CoV-2. These are the four target domains that are targeted the most.

## Usage Notes

To serve the scientific community, a professional and user-friendly web interface was implemented for ACovPepDB. The primary function of the database was embedded in five web pages: browse page, search page, download page, BLAST search page, and peptide three-dimensional structure visualization page.

### Browse

**Fig. 3:**
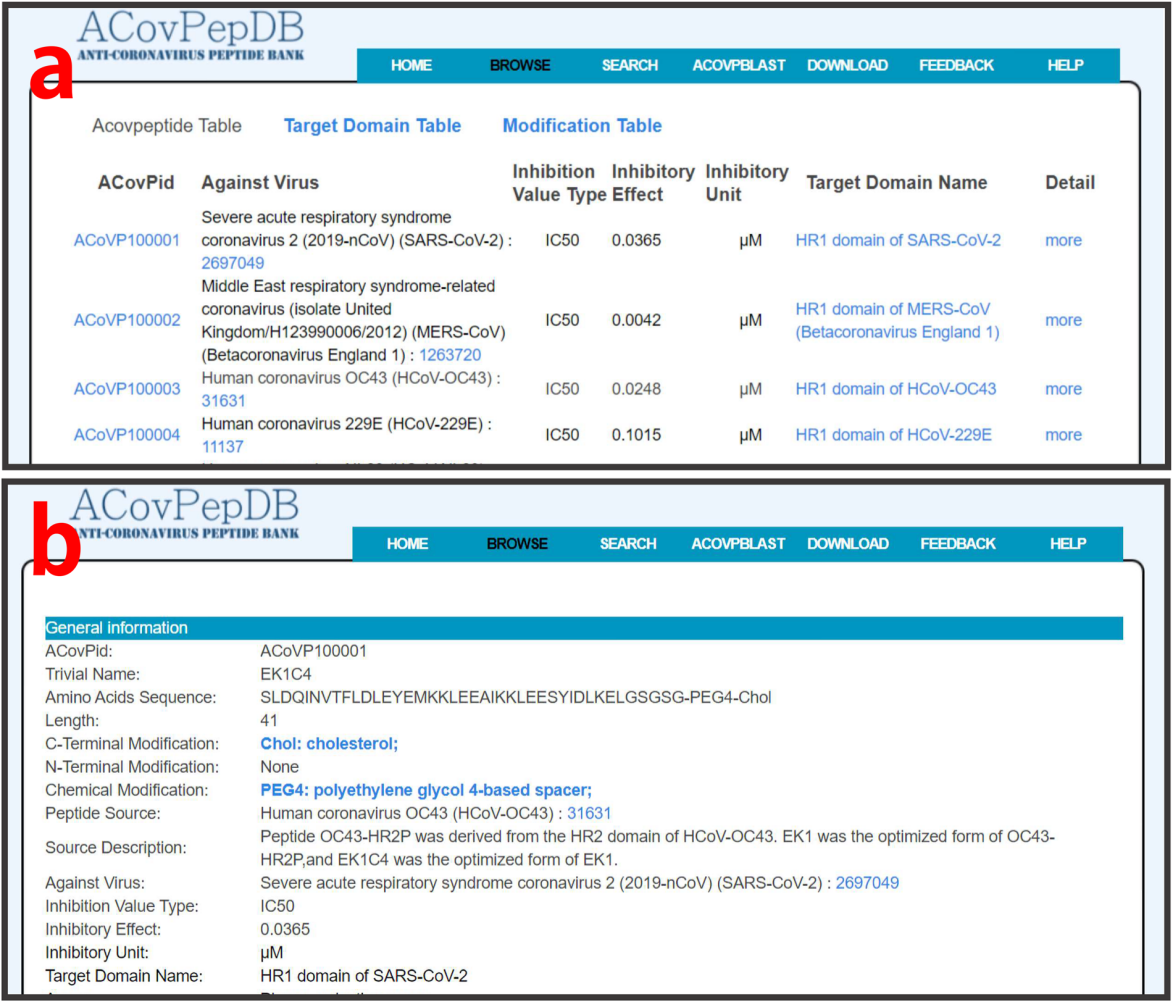
(**a**) Brief browsing page of the ACovpeptide table. (**b**) The detailed browsing page of each entry in the ACovpeptide table.

**Fig. 4:**
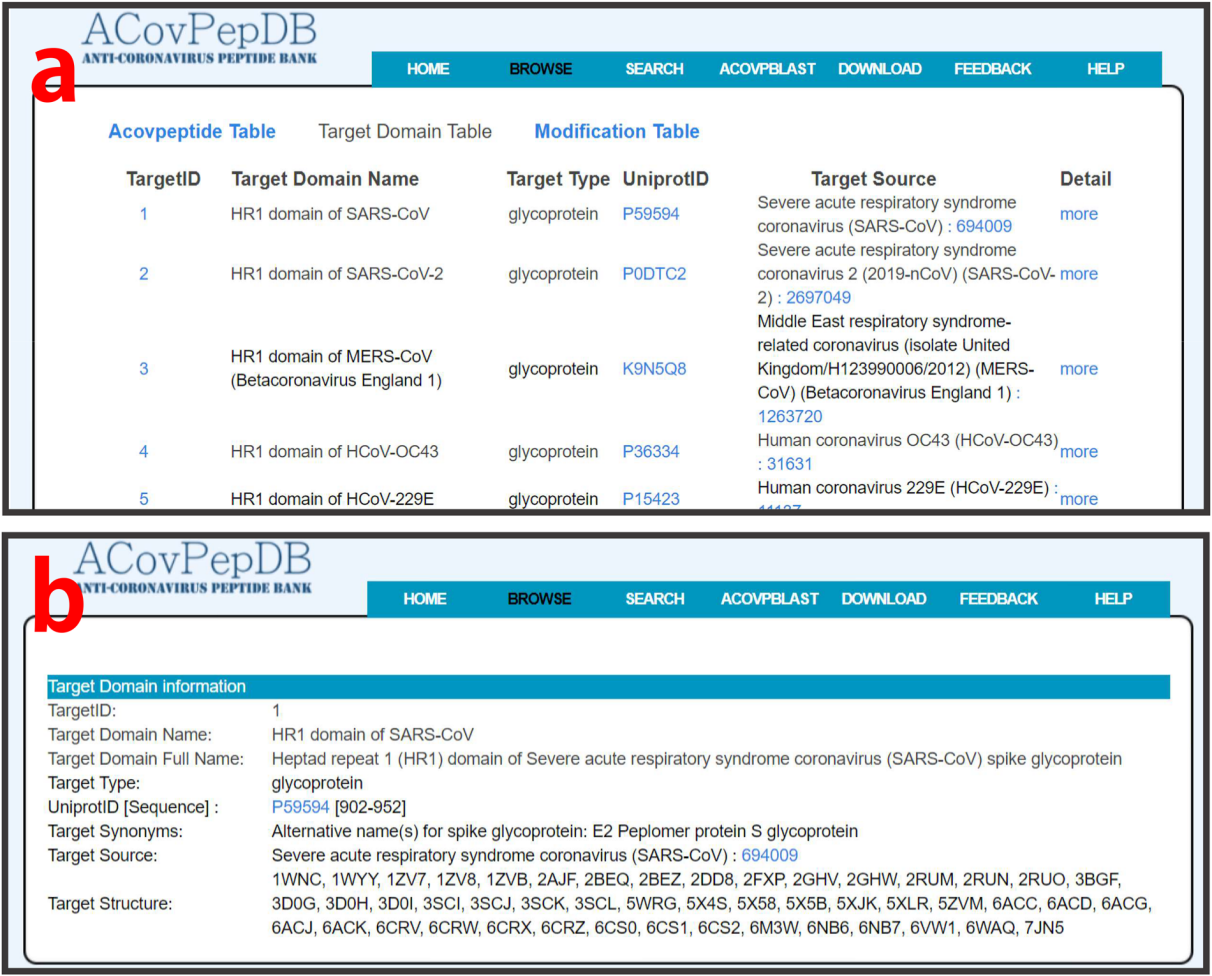
(**a**) Brief browsing page of the Targetdomain table. (**b**) The detailed browsing page of each entry in the Targetdomain table.

**Fig. 5:**
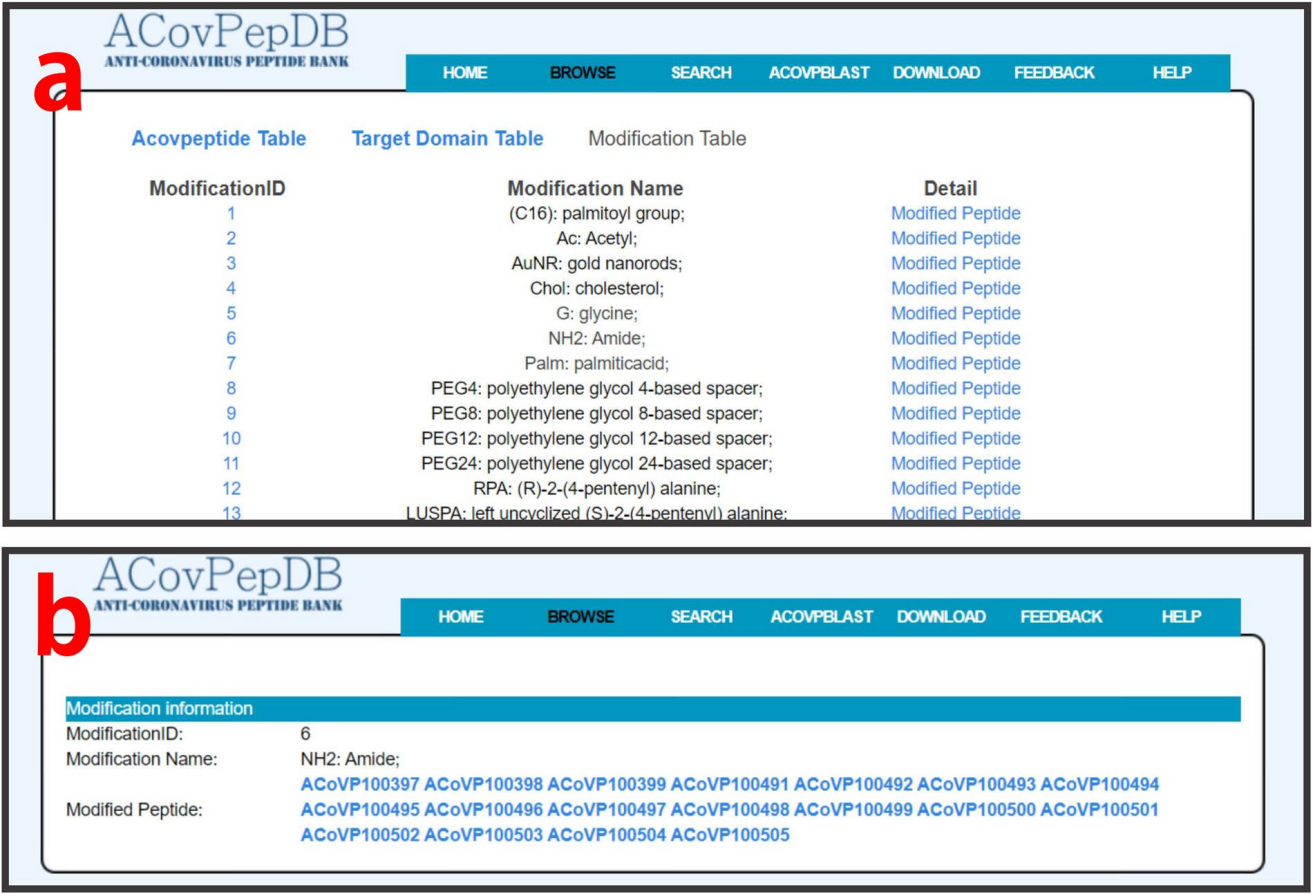
(**a**) Brief browsing page of the Modification table. (**b**) The detailed browsing page of each entry in the Modification table.

Tables “ACovpeptide”, “Targetdomain” and “Modification” can be conveniently explored on the “Browse” page. Users can freely switch between the three tables. By clicking one of the three tables, users firstly access a summary table of each entry. For table “ACovpeptide”, important fields, including ACovPid, against virus, target domain, inhibitory effect, and links guiding to the “Detail” page, are provided in the brief browsing table (Fig. 3a,b). For table “Targetdomain”, the compact browsing table displays TargetID, target domain name, target type, UniprotID, target source, and links to the “Detail” page (Fig. 4a). Users can retrieve detailed information of each entry on the “Detail” page by clicking “more” (Fig. 4b). The “Modification” table allows users to retrieve each type of modification in ACovPepDB (Fig. 5a). Each entry in this table contains three fields: ModificationID, Modification Name, Modified Peptide (Fig. 5b). The “Modified Peptide” field provides links to ACovPs modified by this type of modification.

### Search

**Fig. 6:**
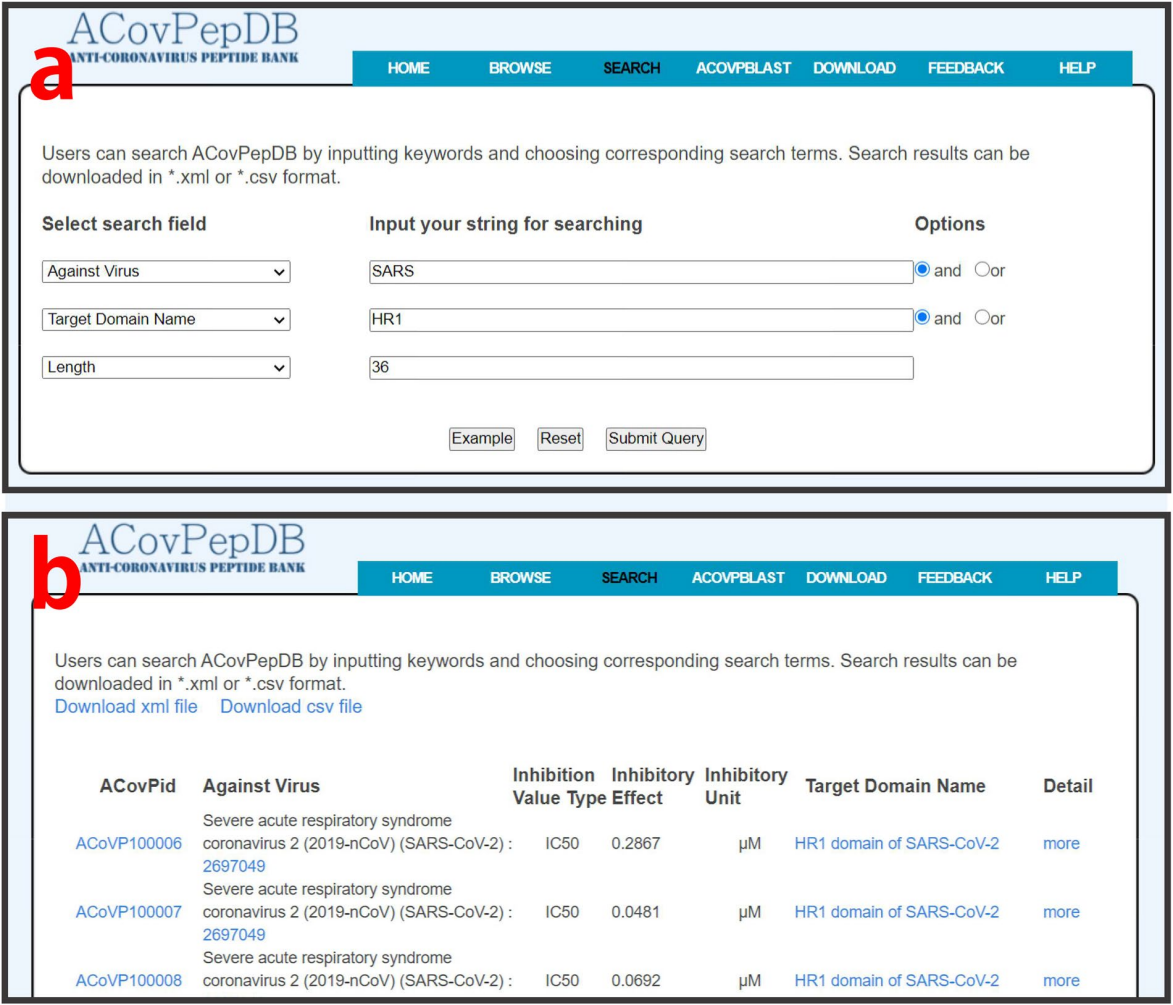
Overview of search page of ACovPepDB. (**a**) Compound search by using Boolean expression. (**b**) Search result page.

The “Search” page can be reached by clicking “Search” at the top of the web interface. Users can easily use simple or compound search methods to precisely fetch data from ACovPepDB. For a simple search, users can search “ACovpeptide”, “Targetdomain” or “Modification” table by inputting any keywords and choosing search terms, such as ACovPid, sequence, peptide source, target domain name, etc. In the compound search module, users can use a Boolean expression to submit a query (e.g., AND, OR) (Fig. 6a). Such a query returns all entries related to the keyword (Fig. 6b). Of course, ACovPepDB also provides a fuzzy search function. This function allows users to search entries with unclear search fields. The search engine will search corresponding data fields to return a series of entries that contain the input keyword.

### BLAST search

In addition to the search system, ACovPepDB also has a BLAST search function (Supplementary Figure S1a). The “ACovPBLAST” provides users with the ability to perform similar searches based on BLASTP (version 2.2.31+)^32^. This function outputs the ACovPs in ACovPepDB similar to the query peptides, and generates a report with multiple output parameters (Score, E Value, Identities, etc.) (Supplementary Figure S1b). ACovPBLAST also allows users to customize the input parameters (for example, expect value, optimized parameters for short peptide or not).

### Peptide three-dimensional structure browse function

ACovPepDB provides an interactive structure viewer for three-dimensional structures of peptides. Users can visualize the three-dimensional structures of ACovPs by clicking the model link in field “3D structure” of table “ACovpeptide”. The structure browse page integrates a plurality of features of the JSmol applet (e.g., set color style, view type, and model type, etc.) and displays the three-dimensional structure of the ACovP in a JSmol window (Supplementary Figure S2).

### Download

As a public and free database, ACovPepDB provides a page for users to download the complete dataset. The database also provides a custom solution for users to download all search result data when they use the “Search” function. ACovPepDB allows batch data to be downloaded as xml or csv format files. The “download” page also provides links for downloading the database in its entirety or data from a single database table (Table “Acovpeptide”, Table “Targetdomain”, Table “Modification”.

## Supplementary information

Supplementary information

## Data Availability

The source code of the ACovPepDB web interface has been shared on GitHub and Gitee.

## References

[1] Zhou P (2020). A pneumonia outbreak associated with a new coronavirus of probable bat origin. Nature.

[2] Chen B (2020). Overview of lethal human coronaviruses. Signal Transduct Target Ther.

[3] Jung K, Saif LJ, Wang Q (2020). Porcine epidemic diarrhea virus (PEDV): An update on etiology, transmission, pathogenesis, and prevention and control. Virus Res.

[4] Singh AK, Singh A, Singh R, Misra A (2021). Molnupiravir in COVID-19: A systematic review of literature. Diabetes Metab Syndr.

[5] Gupta A (2021). Early Treatment for Covid-19 with SARS-CoV-2 Neutralizing Antibody Sotrovimab. The New England journal of medicine.

[6] Deeks ED (2021). Casirivimab/Imdevimab: First Approval. Drugs.

[7] Ju B (2020). Human neutralizing antibodies elicited by SARS-CoV-2 infection. Nature.

[8] Wu A (2020). Genome Composition and Divergence of the Novel Coronavirus (2019-nCoV) Originating in China. Cell Host Microbe.

[9] Liu STH (2020). Convalescent plasma treatment of severe COVID-19: a propensity score-matched control study. Nature medicine.

[10] Arabi Y (2015). Feasibility, safety, clinical, and laboratory effects of convalescent plasma therapy for patients with Middle East respiratory syndrome coronavirus infection: a study protocol. SpringerPlus.

[11] Sakamoto S, Tanaka H, Morimoto S (2015). Towards the prophylactic and therapeutic use of human neutralizing monoclonal antibodies for Middle East respiratory syndrome coronavirus (MERS-CoV). Annals of translational medicine.

[12] Cohen MS (2021). Monoclonal Antibodies to Disrupt Progression of Early Covid-19 Infection. The New England journal of medicine.

[13] Xie Y (2020). Effect of regular intravenous immunoglobulin therapy on prognosis of severe pneumonia in patients with COVID-19. The Journal of infection.

[14] Hartung HP (2009). Clinical applications of intravenous immunoglobulins (IVIg)–beyond immunodeficiencies and neurology. Clinical and experimental immunology.

[15] Janik, E., Niemcewicz, M., Podogrocki, M., Saluk-Bijak, J. & Bijak, M. Existing Drugs Considered as Promising in COVID-19 Therapy. *Int J Mol Sci* **22**, 5434,10.3390/ijms22115434 (2021). 10.3390/ijms22115434 PMC8196765 34063964

[16] Huang L (2020). Progress in the Research and Development of Anti-COVID-19 Drugs. Frontiers in public health.

[17] Mustafa S, Balkhy H, Gabere MN (2018). Current treatment options and the role of peptides as potential therapeutic components for Middle East Respiratory Syndrome (MERS): A review. J Infect Public Health.

[18] Heydari H (2021). Antiviral peptides against Coronaviridae family: A review. Peptides.

[19] Vilas Boas LCP, Campos ML, Berlanda RLA, de Carvalho Neves N, Franco OL (2019). Antiviral peptides as promising therapeutic drugs. Cell Mol Life Sci.

[20] Rezende SB, Oshiro KGN, Júnior NGO, Franco OL, Cardoso MH (2021). Advances on chemically modified antimicrobial peptides for generating peptide antibiotics. Chemical communications (Cambridge, England).

[21] Zheng BJ (2005). Synthetic peptides outside the spike protein heptad repeat regions as potent inhibitors of SARS-associated coronavirus. Antiviral therapy.

[22] Li Q (2011). Virucidal activity of a scorpion venom peptide variant mucroporin-M1 against measles, SARS-CoV and influenza H5N1 viruses. Peptides.

[23] Xia, S. *et al*. Peptide-Based Membrane Fusion Inhibitors Targeting HCoV-229E Spike Protein HR1 and HR2 Domains. *Int J Mol Sci* **19**, 487, 10.3390/ijms19020487 (2018). 10.3390/ijms19020487 PMC5855709 29415501

[24] Xia S (2019). A pan-coronavirus fusion inhibitor targeting the HR1 domain of human coronavirus spike. Science advances.

[25] Wang G, Li X, Wang Z (2016). APD3: the antimicrobial peptide database as a tool for research and education. Nucleic Acids Res.

[26] Jhong JH (2019). dbAMP: an integrated resource for exploring antimicrobial peptides with functional activities and physicochemical properties on transcriptome and proteome data. Nucleic Acids Res.

[27] Kang X (2019). DRAMP 2.0, an updated data repository of antimicrobial peptides. Sci Data.

[28] Kaushik AC (2021). CoronaPep: An Anti-Coronavirus Peptide Generation Tool. IEEE/ACM Trans Comput Biol Bioinform.

[29] Pang Y, Wang Z, Jhong JH, Lee TY (2021). Identifying anti-coronavirus peptides by incorporating different negative datasets and imbalanced learning strategies. Brief Bioinform.

[30] Qureshi A, Thakur N, Tandon H, Kumar M (2014). AVPdb: a database of experimentally validated antiviral peptides targeting medically important viruses. Nucleic Acids Res.

[31] Wang, F. *et al*. DPL: a comprehensive database on sequences, structures, sources and functions of peptide ligands. *Database (Oxford)* **2020**, baaa089, 10.1093/database/baaa089 (2020). 10.1093/database/baaa089 PMC7678785 33216893

[32] Camacho C (2009). BLAST+: architecture and applications. BMC bioinformatics.

[33] Lamiable A (2016). PEP-FOLD3: faster de novo structure prediction for linear peptides in solution and in complex. Nucleic Acids Res.

[34] Kelley LA, Mezulis S, Yates CM, Wass MN, Sternberg MJ (2015). The Phyre2 web portal for protein modeling, prediction and analysis. Nature protocols.

[35] *Anti-Coronavirus Peptide Database* http://i.uestc.edu.cn/ACovPepDB/download.html (2022)

[36] Zhang Q (2022). Figshare.

