# Supplementary material for: A database of anti-coronavirus peptides
Source: Sci Data. 2022 Jun 13;9:294. doi: 10.1038/s41597-022-01394-3 (PMC9192597; doi:10.1038/s41597-022-01394-3)
Supplement: Supplementary file 1 — Supplementary information [file 41597_2022_1394_MOESM1_ESM.pdf]

Supplementary information

Table of contents

Supplementary Source Article	Source articles containing ACovPs data
Supplementary Figure S1	(a) The “ACovPBLAST” page in ACovPepDB. (b) The “ACovPBLAST” result page.
Supplementary Figure S2	The web page of “Peptide three-dimensional structure browse function”.

Supplementary Source Article References

[1] O'Keefe, B. R. et al. Broad-spectrum in vitro activity and in vivo efficacy of the antiviral protein griffithsin against emerging viruses of the family Coronaviridae. Journal of virology 84, 2511-2521, doi:10.1128/jvi.02322-09 (2010).

[2] Zhao, H. et al. A broad-spectrum virus- and host-targeting peptide against respiratory viruses including influenza virus and SARS-CoV-2. Nature communications 11, 4252, doi:10.1038/s41467-020-17986-9 (2020).

[3] Wang, C. et al. Combining a Fusion Inhibitory Peptide Targeting the MERS-CoV S2 Protein HR1 Domain and a Neutralizing Antibody Specific for the S1 Protein Receptor-Binding Domain (RBD) Showed Potent Synergism against Pseudotyped MERS-CoV with or without Mutations in RBD. Viruses 11, doi:10.3390/v11010031 (2019).

[4] Wang, Y. et al. Coronavirus nsp10/nsp16 Methyltransferase Can Be Targeted by nsp10-Derived Peptide In Vitro and In Vivo To Reduce Replication and Pathogenesis. *Journal of virology* **89**, 8416-8427, doi:10.1128/jvi.00948-15 (2015).

[5] Cao, L. *et al.* De novo design of picomolar SARS-CoV-2 miniprotein inhibitors. *Science (New York, N.Y.)* **370**, 426-431, doi:10.1126/science.abd9909 (2020).

[6] Wang, C. *et al.* De Novo Design of α-Helical Lipopeptides Targeting Viral Fusion Proteins: A Promising Strategy for Relatively Broad-Spectrum Antiviral Drug Discovery. *Journal of medicinal chemistry* **61**, 8734-8745, doi:10.1021/acs.jmedchem.8b00890 (2018).

[7] Ho, T. Y. *et al.* Design and biological activities of novel inhibitory peptides for SARS-CoV spike protein and angiotensin-converting enzyme 2 interaction. *Antiviral research* **69**, 70-76, doi:10.1016/j.antiviral.2005.10.005 (2006).

[8] Zhu, Y., Yu, D., Yan, H., Chong, H. & He, Y. Design of Potent Membrane Fusion Inhibitors against SARS-CoV-2, an Emerging Coronavirus with High Fusogenic Activity. *Journal of virology* **94**, doi:10.1128/jvi.00635-20 (2020).

[9] Huang, Y. *et al.* Design, synthesis and screening of antisense peptide based combinatorial peptide libraries towards an aromatic region of SARS-CoV. *Journal of molecular recognition : JMR* **21**, 122-131, doi:10.1002/jmr.880 (2008).

[10] Wang, C. *et al.* Discovery of Hydrocarbon-Stapled Short α-Helical Peptides as Promising Middle East Respiratory Syndrome Coronavirus (MERS-CoV) Fusion Inhibitors. *Journal of medicinal chemistry* **61**, 2018-2026, doi:10.1021/acs.jmedchem.7b01732 (2018).

[11] Kandeel, M. *et al.* Discovery of New Fusion Inhibitor Peptides against SARS-CoV-2 by Targeting the Spike S2 Subunit. *Biomolecules & therapeutics* **29**, 282-289, doi:10.4062/biomolther.2020.201 (2021).

[12] Zhu, J. *et al.* Following the rule: formation of the 6-helix bundle of the fusion core from severe acute respiratory syndrome coronavirus spike protein and identification of potent peptide inhibitors. *Biochemical and biophysical research communications* **319**, 283-288, doi:10.1016/j.bbrc.2004.04.141 (2004).

[13] Chu, L. H. *et al.* Fusion core structure of the severe acute respiratory syndrome coronavirus (SARS-CoV): in search of potent SARS-CoV entry inhibitors. *Journal of cellular biochemistry* **104**, 2335-2347, doi:10.1002/jcb.21790 (2008).
[14] Xia, S. *et al.* Fusion mechanism of 2019-nCoV and fusion inhibitors targeting HR1 domain in spike protein. *Cellular & molecular immunology* **17**, 765-767, doi:10.1038/s41423-020-0374-2 (2020).
[15] Ujike, M. *et al.* Heptad repeat-derived peptides block protease-mediated direct entry from the cell surface of severe acute respiratory syndrome coronavirus but not entry via the endosomal pathway. *Journal of virology* **82**, 588-592, doi:10.1128/jvi.01697-07 (2008).
[16] Struck, A. W., Axmann, M., Pfefferle, S., Drosten, C. & Meyer, B. A hexapeptide of the receptor-binding domain of SARS corona virus spike protein blocks viral entry into host cells via the human receptor ACE2. *Antiviral research* **94**, 288-296, doi:10.1016/j.antiviral.2011.12.012 (2012).
[17] Karoyan, P. *et al.* Human ACE2 peptide-mimics block SARS-CoV-2 pulmonary cells infection. *Communications biology* **4**, 197, doi:10.1038/s42003-021-01736-8 (2021).
[18] Wang, C. *et al.* Human Cathelicidin Inhibits SARS-CoV-2 Infection: Killing Two Birds with One Stone. *ACS infectious diseases* **7**, 1545-1554, doi:10.1021/acsinfecdis.1c00096 (2021).
[19] Liu, I. J. *et al.* Identification of a minimal peptide derived from heptad repeat (HR) 2 of spike protein of SARS-CoV and combination of HR1-derived peptides as fusion inhibitors. *Antiviral research* **81**, 82-87, doi:10.1016/j.antiviral.2008.10.001 (2009).
[20] Guo, Y. *et al.* Identification of a new region of SARS-CoV S protein critical for viral entry. *Journal of molecular biology* **394**, 600-605, doi:10.1016/j.jmb.2009.10.032 (2009).
[21] Sun, Y., Zhang, H., Shi, J., Zhang, Z. & Gong, R. Identification of a Novel Inhibitor against Middle East Respiratory Syndrome Coronavirus. *Viruses* **9**, doi:10.3390/v9090255 (2017).
[22] Han, D. P., Penn-Nicholson, A. & Cho, M. W. Identification of critical determinants on ACE2 for SARS-CoV entry and development of a potent entry inhibitor. *Virology* **350**, 15-25, doi:10.1016/j.virol.2006.01.029 (2006).
[23] Ling, R. *et al.* In silico design of antiviral peptides targeting the spike protein of SARS-CoV-2. *Peptides* **130**, 170328, doi:10.1016/j.peptides.2020.170328 (2020).
[24] Outlaw, V. K. *et al.* Inhibition of Coronavirus Entry In Vitro and Ex Vivo by a Lipid-Conjugated Peptide Derived from the SARS-CoV-2 Spike Glycoprotein HRC Domain. *mBio* **11**, doi:10.1128/mBio.01935-20 (2020).
[25] Xia, S. *et al.* Inhibition of SARS-CoV-2 (previously 2019-nCoV) infection by a highly potent pan-coronavirus fusion inhibitor targeting its spike protein that harbors a high capacity to mediate membrane fusion. *Cell Res* **30**, 343-355, doi:10.1038/s41422-020-0305-x (2020).

[26] Sainz, B., Jr. *et al.* Inhibition of severe acute respiratory syndrome-associated coronavirus (SARS-CoV) infectivity by peptides analogous to the viral spike protein. *Virus Res* **120**, 146-155, doi:10.1016/j.virusres.2006.03.001 (2006).
[27] Liu, S. *et al.* Interaction between heptad repeat 1 and 2 regions in spike protein of SARS-associated coronavirus: implications for virus fusogenic mechanism and identification of fusion inhibitors. *Lancet (London, England)* **363**, 938-947, doi:10.1016/s0140-6736(04)15788-7 (2004).
[28] Odolczyk, N., Marzec, E., Winiewska-Szajewska, M., Poznański, J. & Zielenkiewicz, P. Native Structure-Based Peptides as Potential Protein-Protein Interaction Inhibitors of SARS-CoV-2 Spike Protein and Human ACE2 Receptor. *Molecules (Basel, Switzerland)* **26**, doi:10.3390/molecules26082157 (2021).
[29] Huang, X. *et al.* Novel Gold Nanorod-Based HR1 Peptide Inhibitor for Middle East Respiratory Syndrome Coronavirus. *ACS applied materials & interfaces* **11**, 19799-19807, doi:10.1021/acsami.9b04240 (2019).
[30] Zhao, H. *et al.* A novel peptide with potent and broad-spectrum antiviral activities against multiple respiratory viruses. *Scientific reports* **6**, 22008, doi:10.1038/srep22008 (2016).
[31] Xia, S. *et al.* A pan-coronavirus fusion inhibitor targeting the HR1 domain of human coronavirus spike. *Science advances* **5**, eaav4580, doi:10.1126/sciadv.aav4580 (2019).
[32] Xia, S. *et al.* Peptide-Based Membrane Fusion Inhibitors Targeting HCoV-229E Spike Protein HR1 and HR2 Domains. *Int J Mol Sci* **19**, doi:10.3390/ijms19020487 (2018).
[33] Liu, I. J., Tsai, W. T., Hsieh, L. E. & Chueh, L. L. Peptides corresponding to the predicted heptad repeat 2 domain of the feline coronavirus spike protein are potent inhibitors of viral infection. *PloS one* **8**, e82081, doi:10.1371/journal.pone.0082081 (2013).
[34] Ren, X., Liu, B., Yin, J., Zhang, H. & Li, G. Phage displayed peptides recognizing porcine aminopeptidase N inhibit transmissible gastroenteritis coronavirus infection in vitro. *Virology* **410**, 299-306, doi:10.1016/j.virol.2010.11.014 (2011).
[35] Xia, S. *et al.* Potent MERS-CoV Fusion Inhibitory Peptides Identified from HR2 Domain in Spike Protein of Bat Coronavirus HKU4. *Viruses* **11**, doi:10.3390/v11010056 (2019).
[36] Channappanavar, R. *et al.* Protective Effect of Intranasal Regimens Containing Peptidic Middle East Respiratory Syndrome Coronavirus Fusion Inhibitor Against MERS-CoV Infection. *The Journal of infectious diseases* **212**, 1894-1903, doi:10.1093/infdis/jiv325 (2015).
[37] Larue, R. C. *et al.* Rationally Designed ACE2-Derived Peptides Inhibit SARS-CoV-2. *Bioconjugate chemistry* **32**, 215-223, doi:10.1021/acs.bioconjchem.0c00664 (2021).
[38] Hu, H. *et al.* Screening and identification of linear B-cell epitopes and entry-blocking peptide of severe acute respiratory syndrome (SARS)-associated coronavirus

using synthetic overlapping peptide library. *Journal of combinatorial chemistry* **7**, 648-656, doi:10.1021/cc0500607 (2005).
[39] Bosch, B. J. *et al.* Severe acute respiratory syndrome coronavirus (SARS-CoV) infection inhibition using spike protein heptad repeat-derived peptides. *Proceedings of the National Academy of Sciences of the United States of America* **101**, 8455-8460, doi:10.1073/pnas.0400576101 (2004).
[40] Ke, M. *et al.* Short peptides derived from the interaction domain of SARS coronavirus nonstructural protein nsp10 can suppress the 2'-O-methyltransferase activity of nsp10/nsp16 complex. *Virus Res* **167**, 322-328, doi:10.1016/j.virusres.2012.05.017 (2012). [41] Curreli, F. *et al.* Stapled Peptides Based on Human Angiotensin-Converting Enzyme 2 (ACE2) Potently Inhibit SARS-CoV-2 Infection In Vitro. *mBio* **11**, doi:10.1128/mBio.02451-20 (2020).
[42] Sun, H. *et al.* Structural basis of HCoV-19 fusion core and an effective inhibition peptide against virus entry. *Emerging microbes & infections* **9**, 1238-1241, doi:10.1080/22221751.2020.1770631 (2020).
[43] Gao, J. *et al.* Structure of the fusion core and inhibition of fusion by a heptad repeat peptide derived from the S protein of Middle East respiratory syndrome coronavirus. *Journal of virology* **87**, 13134-13140, doi:10.1128/jvi.02433-13 (2013).
[44] Lu, L. *et al.* Structure-based discovery of Middle East respiratory syndrome coronavirus fusion inhibitor. *Nature communications* **5**, 3067, doi:10.1038/ncomms4067 (2014).
[45] Wang, C. *et al.* Supercoiling Structure-Based Design of a Trimeric Coiled-Coil Peptide with High Potency against HIV-1 and Human β-Coronavirus Infection. *Journal of medicinal chemistry* **65**, 2809-2819, doi:10.1021/acs.jmedchem.1c00258 (2022).
[46] Yuan, K. *et al.* Suppression of SARS-CoV entry by peptides corresponding to heptad regions on spike glycoprotein. *Biochemical and biophysical research communications* **319**, 746-752, doi:10.1016/j.bbrc.2004.05.046 (2004).
[47] Gan, Y. R. *et al.* Synthesis and activity of an octapeptide inhibitor designed for SARS coronavirus main proteinase. *Peptides* **27**, 622-625, doi:10.1016/j.peptides.2005.09.006 (2006).
[48] Lu, W. *et al.* Synthetic peptides derived from SARS coronavirus S protein with diagnostic and therapeutic potential. *FEBS letters* **579**, 2130-2136, doi:10.1016/j.febslet.2005.02.070 (2005).
[49] Zheng, B. J. *et al.* Synthetic peptides outside the spike protein heptad repeat regions as potent inhibitors of SARS-associated coronavirus. *Antiviral therapy* **10**, 393-403 (2005).
[50] Maas, M. N., Hintzen, J. C. J., Löffler, P. M. G. & Mecinović, J. Targeting SARS-CoV-2 spike protein by stapled hACE2 peptides. *Chemical communications (Cambridge, England)* **57**, 3283-3286, doi:10.1039/d0cc08387a (2021).
[51] Liao, Y., Zhang, S. M., Neo, T. L. & Tam, J. P. Tryptophan-dependent membrane interaction and heteromerization with the internal fusion peptide by the membrane proximal external region of SARS-CoV spike protein. *Biochemistry* **54**, 1819-1830, doi:10.1021/bi501352u (2015).

[52] Li, Q. *et al*. Virucidal activity of a scorpion venom peptide variant mucroporin-M1 against measles, SARS-CoV and influenza H5N1 viruses. *Peptides* **32**, 1518-1525, doi:10.1016/j.peptides.2011.05.015 (2011).

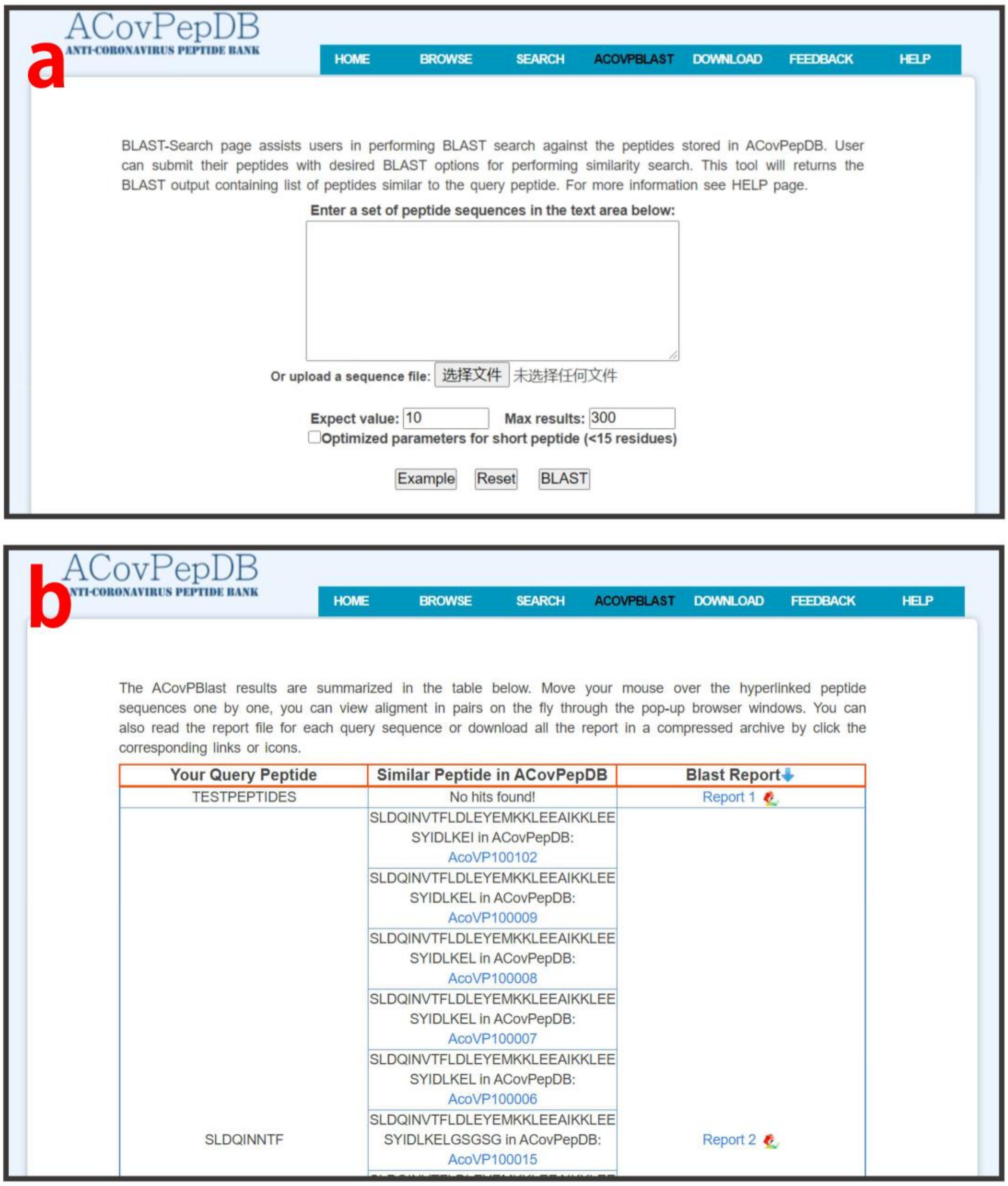

Supplementary Figure S1 The ACovPBLAST tool.

(a) The ACovPBLAST input page. (b) The ACovPBLAST result page.

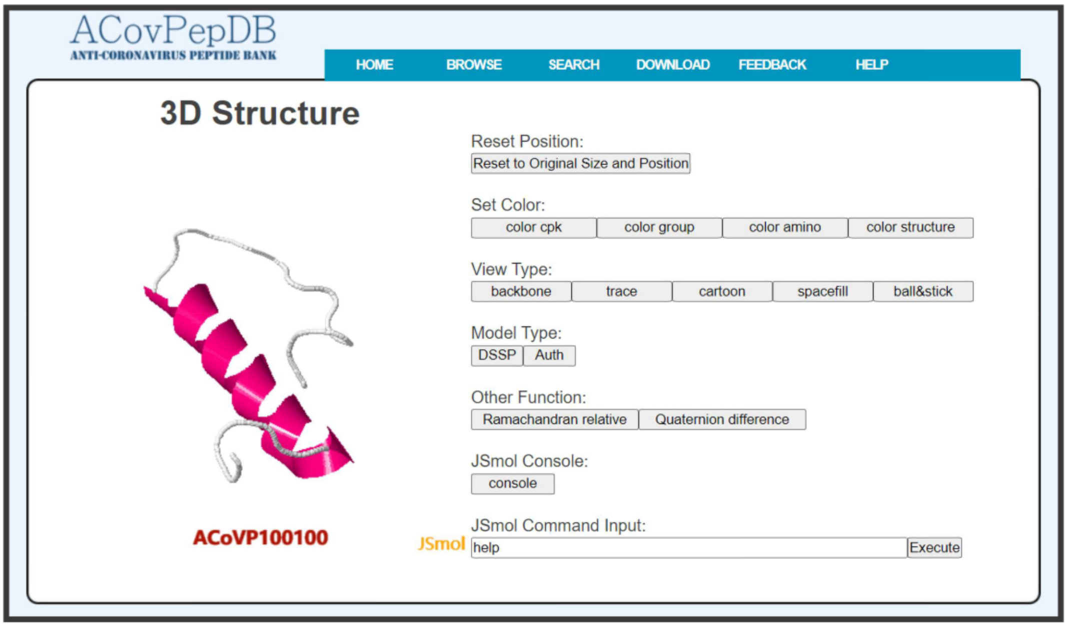

Supplementary Figure S2

Peptide three-dimensional structure visualized using the JSmol program
